# Design of a novel multiepitope vaccine against *Chlamydia pneumoniae* using the extracellular protein as a target

**DOI:** 10.1038/s41598-023-42222-x

**Published:** 2023-09-12

**Authors:** Xiaomei Guo, Xiaohong Pan, Qiangming Sun, Yunzhang Hu, Jiandong Shi

**Affiliations:** 1 Yunnan Provincial Key Laboratory of Vector-Borne Diseases Control and Research, Institute of Medical Biology, Chinese Academy of Medical Sciences and Peking Union Medical College, 935 Jiaoling Road, Kunming, 650118 Yunnan China; 2 National Kunming High-Level Biosafety Primate Research Center, Institute of Medical Biology, Chinese Academy of Medical Sciences and Peking Union Medical College, Kunming, Yunnan China; 3 Kunming Medical University, Kunming, Yunnan China

**Keywords:** Computational biology and bioinformatics, Immunology, Microbiology

## Abstract

*Chlamydia pneumoniae* (*C. pneumoniae*) infection in humans is universal and causes various respiratory infectious diseases, making a safe and effective preventive vaccine essential. In this study, a multi-epitope vaccine with CTLA-4 extracellular structure was constructed by an immunoinformatics approach. Since MOMP protein is the major extracellular protein in *C. pneumoniae* and has good immunogenicity and high conservation, we selected the MOMP protein of *C. pneumoniae* as the antigen target, predicted the T and B cell epitopes of the MOMP protein and then connected the CTLA-4 extracellular structure with the predicted dominant epitopes by various linkers to construct a multi-epitope vaccine. The biochemical characterization of the multi-epitope vaccine showed its immunogenicity and anti-allergic properties. The tertiary structure of this vaccine, along with molecular docking, molecular dynamics simulation, and principal component analysis, showed that the multi-epitope vaccine structure interacted with B7 (B7-1, B7-2) and toll-like receptors (TLR-2, TLR-4). Ultimately, the vaccine was cloned and effectively expressed in silico on an insect baculovirus expression vector (pFastBac1). These analyses showed that the designed vaccine could potentially target antigen-presenting cells and was immune to *C. pneumoniae*, which provided novel strategies for developing the vaccine.

## Introduction

*Chlamydia* is a class of prokaryotic cellular microorganisms with a unique developmental cycle and specialized intracellular parasitism with two developmental cycles: cthe infectious elementary body (EB) and the reticulate body (RB). The EB is the extracellular stage of infection, and the RB is the metabolically active intracellular replication^1^. The main species of *Chlamydia* that are closely related to humans are *Chlamydia trachomatis* (CT), *Chlamydia psittaci* (Cps), and *Chlamydia pneumoniae* (*C. penumoniae*; Cpn)^2^. The specialized intracellular Gram-negative bacteria of the genus *Chlamydia* can cause ocular, genital, and respiratory infections and have considerable public health implications. It was estimated that > 60% of individuals in the majority of American, European, and Asian countries had been exposed to *C. pneumoniae*, causing widespread respiratory illnesses in humans^3^.

A specific feature of *C. pneumoniae* is its ability to spread from the pulmonary system via peripheral blood monocytes and localize in several extra-pulmonary tissues, including arteries, joints, bone, and the central nervous system^4^. *C. pneumoniae* mainly causes atypical pneumonia and respiratory infections in adults and adolescents. It is responsible for 10% of community-acquired pneumoniae and 5% of bronchitis, pharyngitis, and sinusitis^5^ and has also been found to be closely associated with the development of cardiovascular disease, Alzheimer’s disease, and asthma^6–9^. Therefore, there is an urgent need for rapid diagnosis and the treatment of infection to avoid the problems associated with *C. pneumoniae* infection.

The main drugs used to treat *C. pneumoniae* infections are macrolides, but the unique survival cycle of chlamydia allows *chlamydial* infections to persist in the body, leading to increased macrolide resistance^9^. The current studies on Chlamydia vaccines have shown that a *Chlamydia trachomatis* vaccine with the recombinant antigen CTH522 has entered phase I clinical trials on females as primary subjects and shown a robust immune response ^10^. However, there is no vaccine for human *C. pneumoniae* infection, and no single antigenic epitope has achieved satisfactory results; thus, it can be inferred that a balance of multiple antigenic epitopes is essential^10,11^.

Toll-like receptors (TLRs) were the first protein family to realize Janway's predictions of the defining characteristics of pattern recognition receptors (PRRs), which are purportedly germline-encoded proteins that recognize conserved microbial products (pathogen-associated molecular patterns [PAMPs]) and thereby trigger immune system and host defense-stimulating activities. Moreover, TLR-2 and TLR-4 are the major receptors on the surface of immune cell subsets^12^. It has been shown that TLR-2 and TLR-4 play a key role in mediating the immune response to *Chlamydia pneumoniae* infection^13–15^.

In recent decades, research on *Chlamydia* vaccines has centered on the discovery of an immunogen and protective antigen that can be employed as a subunit and peptide vaccine^16^. Several research conducted on mice and koalas have demonstrated that the main outer membrane protein (MOMP) is a well-known antigenic target and an effective substitute for whole-cell targets^17,18^. Therefore, it was selected as a *Chlamydia* vaccine candidate antigen mainly for the following reasons: (1) MOMP constitutes about 60% of extra-membrane proteins, is expressed in all stages of the *Chlamydia* life cycle, and is relatively conserved across the genus; (2) MOMP is highly immunogenic and elicits T-cell responses and neutralizing antibodies; (3) MOMP is an immunogenic protein recognized during *C. pneumoniae* infection^19,20^.

Cytotoxic T lymphocyte-associated antigen-4 (CTLA-4, CD152) is a membrane glycoprotein produced by activated effector T cells (Teffs) that is involved in the suppression of T cell proliferation, cell cycle progression, and cytokine (IL-2 and IFN-γ) production ^21^. When expressed on activated T cells, CTLA-4 binds with high affinity to CD80 (B7-1) and CD86 (B7-2) molecules present on APCs and delivers negative regulatory signals to T cells^22^. A previous study has shown that fusion expression of the extracellular structural domain of CTLA-4 with the antigen enhances the immunogenicity of the antigen, thus rendering a good immune response to the vaccine^23^. In addition, we can remove the intracellular and transmembrane structural domains and retain the high affinity between the extracellular structural domain of CTLA-4 and the B7 molecule on antigen-presenting cells (APCs).

Over the past few decades, there has been an unprecedented growth in the development of new vaccines. Because of this, vaccine-preventable illnesses, fewer injections, and highly safe and pure vaccines have been prevented. Reverse vaccinology, structural biology, and systems biology are some of the novel discovery methodologies that offer the potential to develop new vaccines for various illnesses^24^. We used an immunoinformatics approach to predict the B- and T-cell epitopes of MOMP, linked the extracellular structure of CTLA-4 with antigenic epitopes using linker sequences to construct a multi-epitope vaccine, and assessed the biochemical properties, antigenicity and allergenicity of the vaccine. Then, the interactions between the multi-epitope vaccine and human B7-1, B7-2, TLR-2, and TLR-4 were evaluated using molecular docking, molecular dynamics (MD) simulation, and principal component analysis (PCA). Finally, vaccine expression vector was constructed using in silico cloning to provide this vector for subsequent in vivo and in vitro animal experiments^25–27^.

## Materials and methods

### Protein sequence retrieval

**Figure 1:**
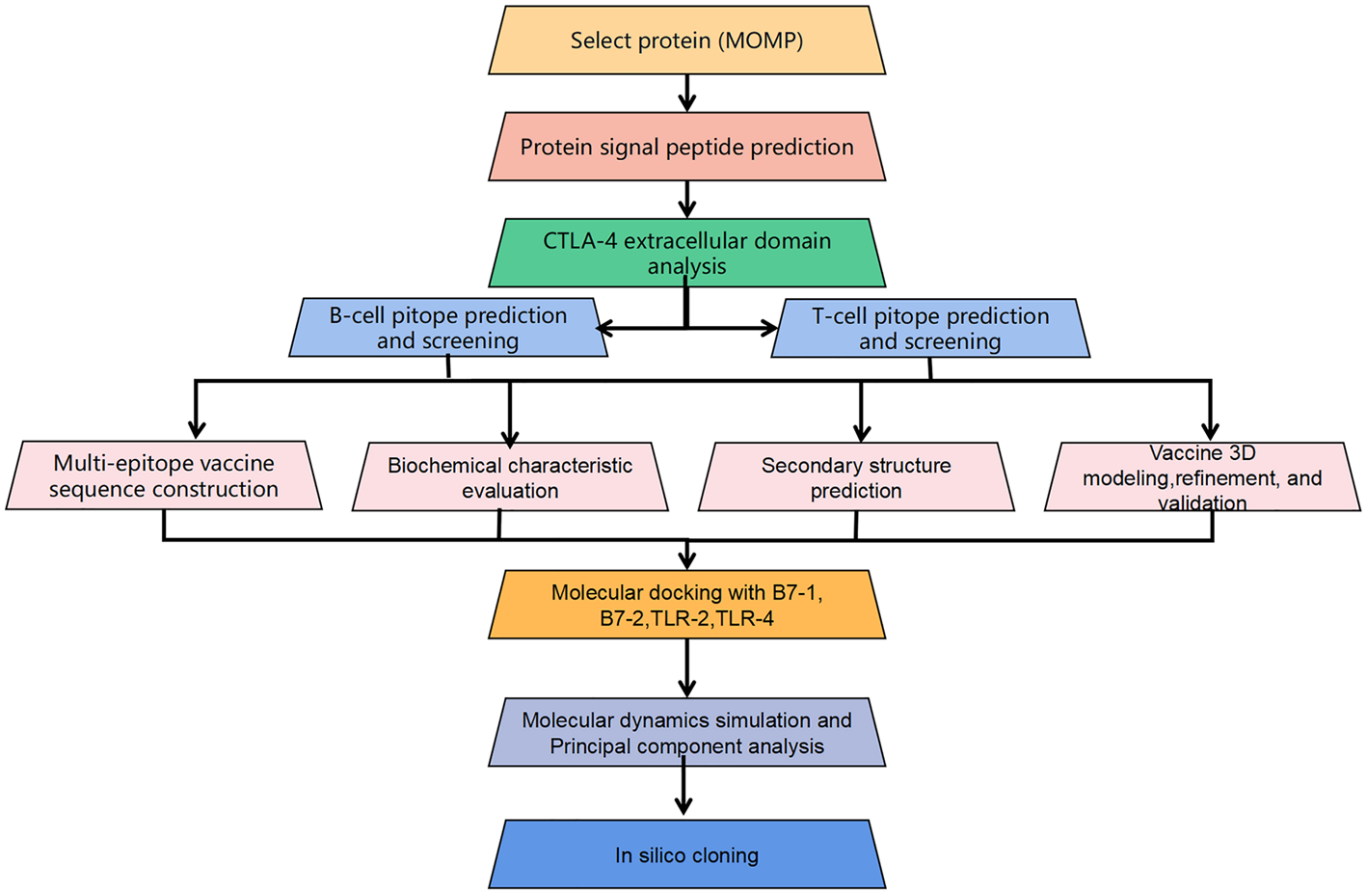
The workflow of this study.

The proteome sequences of *C. pneumoniae* (AAD22492.1) were downloaded from UniProt (https://www.uniprot.org/). The human CTLA-4 amino acid sequence was obtained from the National Center for Biotechnology Information (NCBI). The schematic is illustrated in Fig. 1.

### Protein signal peptide prediction

The newly synthesized proteins are directed to the secretory route by a short (5–30 amino acid) peptide chain known as the signal peptide. The role of the N-terminal signal peptide (SP) is responsible for targeting proteins to the endomembrane system, where they undergo folding and post-translational modifications. Proteins with SPs can either remain in these compartments, insert into the cell membrane or be exported outside the cell^28^. Herein, we used SignalP 6.0 server (https://services.healthtech.dtu.dk/service.php?SignalP) to predict the signal peptide of the major outer membrane protein. The three different forms of prokaryotic SPs may be distinguished using this technique, which can also enhance signal peptide (SP) prediction in all life domains^29^.

### CTLA-4 extracellular domain analysis

CTLA-4, also known as CD152, is a leukocyte differentiation antigen and a transmembrane receptor on T cells. The extracellular structure of CTLA-4 was predicted through DeepTMHMM (https://services.healthtech.dtu.dk/service.php?DeepTMHMM). We also evaluated whether the protein has an extracellular structure and determined whether the protein belongs to the intracellular or extracellular proteins^30^.

### Epitope prediction and screening

BCPREDS(http://ailabprojects1.ist.psu.edu:8080/bcpred/predict.html)^31^ and ABCpred(https://webs.iiitd.edu.in/raghava/abcpred/index.html)^32^ were used to predict the linear B-cell epitopes of MOMP. We set the BCPREDS specificity to 75% and the epitope length to 12; the top 10 hits by score were considered. The ABCpred epitope length was set to 16, and the results were set to the first 10 digits. T-cell epitopes are peptide sequences on the surface of an APC that bind to major histocompatibility complex (MHC) class I and II molecules. The CD4^+^ T-cell epitopes may be improved by the docking of the molecule between the antigen peptide and human leukocyte antigen-II (HLA-II)^33^. HLA class II molecules can bind to the co-receptor CD4 to recognize and purify endogenous antigens. Then, we selected three sets of high-frequency alleles in China, namely HLA-DRB1*15:01, HLA-DRB1*07:01, and HLA-DRB1*11:01^34^. Two types of online software, NetNHCIIpan (https://services.healthtech.dtu.dk/service.php?NetMHCIIpan-4.0)^35^ and SYFPEITHI (http://www.syfpeithi.de/bin/mhcserver.dll/epitopeprediction.htm)^36^ were used to predict the helper T lymphocyte (HTL) epitopes of the selected proteins. The epitope length was set to 15 and the highest three outcomes for each group were used. Ultimately, the common sequences of the two types of software were used to determine the major epitopes of the protein. HLA is the most gene-dense region and plays a critical role in the generation of immune responses ^34^. The frequency of HLA alleles varies among members of various racial groups or members of the same racial group living in different geographical locations. Next, we selected three sets of high-frequency alleles in China, HLA-A*02:01, HLA-A*11:01, and HLA-A*24:02. Cytotoxic T lymphocytes (CTLs) are subsets of white blood cells, which are specific T cells that specialize in secreting various cytokines for immune activity. It has a killing effect on certain viruses, tumor cells, and other antigenic substances, and natural killer cells constitute a defense line of the body’s anti-virus and anti-tumor immunity^37^. As a result, we used EpiJen (http://www.ddgpharmfac.net/epijen/EpiJen/EpiJen.htm)^38^ and NetCTLpan (https://services.healthtech.dtu.dk/service.php?NetCTLpan-1.1)^39^ to predict the CTL epitope. The epitope length was set to 9, the top three in each group were retained, and the overlapping sequences were screened. We used ElliPro online software (http://tools.iedb.org/ellipro/) to predict the conformational epitopes of the MOMP. The ElliPro is a web-based tool for predicting antibody epitopes in protein antigens of a given sequence or structure^40^.

### Vaccine design and prediction of features

In this step, the dominant epitopes of T and B cells were respectively connected with AYY and KK linkers, and used in the construction of multi-epitope vaccines. CTLA-4 is the transmembrane receptor of T cells, binding to B7 molecules to induce cellular responses, involved in immune submodulation. Consequently, the CTLA-4 extracellular structure is connected to the cell epitope with EAKK linker to improve the affinity of immunity. To evaluate the physicochemical properties of vaccines, we used ProtParam (https://web.expasy.org/protparam/), a web-based tool to calculate the molecular weight, theoretical pI, amino acid composition, atomic composition, extinction coefficient, estimated half-life, instability index, aliphatic index, and grand average of hydropathicity (GRAVY)^41^. VaxiJen is the first server for alignment-independent prediction of protective antigens; therefore, we used VaxiJen 2.0 (http://www.ddgpharmfac.net/vaxijen/VaxiJen/VaxiJen.html) to generate vaccine antigenicity^42^, which facilitates antigen classification only by the physicochemical characteristics of the protein without resorting to sequence alignment. To determine the allergenicity of the vaccine, we used Aller-TOP2.0 (http://www.ddg-pharmfac.net/AllerTOP/) for analysis. AllerTOP is a server for the in silico prediction of allergens based on the main physicochemical properties of proteins. The hypersensitivity of the vaccine was predicted by comparison between allergens and non-allergens^43^.

### Prediction of secondary structures

The secondary structure of protein molecules refers to the local spatial structure of a specific peptide chain in protein molecules; the main forms include a helix, β folding, β corners, and irregular curls. The Prabi server (https://npsaprabi.ibcp.fr/cgibin/npsa_automat.pl?page=npsa_sopma.html) is a common secondary structure prediction software. The default window width and similarity threshold were used^44^.

### Vaccine tertiary structure prediction, refinement, and validation

We used trRosetta (https://yanglab.nankai.edu.cn/trRosetta/), a web-based platform for fast and accurate protein structure prediction powered by deep learning. We used the TM score to select the desired tertiary structure of the vaccine, and the final model of the vaccine tertiary structure was provided by trRosetta^45^. After predicting, we employed GalaxyRefine (http://galaxy.seoklab.org/refine) to optimize the tertiary structure of the vaccine. The web server is based on a refinement method successfully tested in CASP10. The strategy uses molecular dynamics simulation to initially rebuild side chains, followed by side-chain repacking and overall structural relaxation. The results of the tertiary structure refinement of vaccines were analyzed by GDT-HA, root mean square deviation (RMSD) and MolProbity score^46^. The Protein Structural Analysis (ProSA) application, which has a large user base, is frequently used in structure prediction and modeling, as well as in improving and validating experimental protein structures. The optimal 3D model of the multi-epitope vaccine was verified using ProSA web (https://prosa.services.came.sbg.ac.at/prosa.php)^47^ and SWISS-MODEL (https://swissmodel.expasy.org/assess)^48^. ProSA is a widely used tool to evaluate the 3D models of protein structures for potential errors.

### Molecular docking

To analyze the interaction of the vaccine with TLR-2, TLR-4 and B7-1 and B7-2, we retrieved and downloaded B7-1 (PDB ID 1DR9), B7-2 (PDB ID 1NCN), TLR-2 (PDB ID 2Z80), and TLR-4 (PDB ID 2Z63) of the 3D models. A tool to computationally build three-dimensional (3D) models of a protein complex structure based on its constituent protein units is provided by protein–protein docking programs. LZerD Web Server (https://lzerd.kiharalab.org/upload/upload/) generates sophisticated protein models by combining shape-based protein surface features and physics-based scoring terms. The molecular docking results were selected by rank-sum (the smaller, the better) score^49^.

### Molecular dynamics simulation

GROMACS 2021.5 software package was used to simulate the protein and ligand molecular dynamics using amber 14 sb force field. Then, by establishing periodic boundary conditions, the TIP3P dominating water model was chosen. Cl ions were added to the protein surface to neutralize the overall charges of the systems. The workflow of molecular dynamics simulation includes four steps: energy minimization, NVT (isothermal-isochoric, represents a certain number of particles, volume, temperature) equilibrium, NPT (isothermal-isobaric, represents a certain number of particles, pressure, temperature) equilibrium, and production dynamics simulation. Firstly, the protein and heavy ligand atoms were bound to minimize the energy of water molecules by 5000 steps using the steepest descent method. The limitations were kept, and a 50,000 step NVT ensemble simulation was run for the entire system. The temperature was 298 K, and the time step was 2 fs. Then, the entire system was subjected to a 50,000 step NPT ensemble simulation at a temperature of 298 K and a time step of 2 fs. Finally, the system was molecularly simulated in the NPT ensemble for 200 ns with a time step of 2 fs. The relevant parameters were examined with the GROMACS software package's module^50^.

### Principal component analysis

Principal component analysis (PCA) was performed in molecular dynamics simulation (MD simulation), in which principle components (PCs) are eigenvectors that specify the motion's direction and eigenvalues specify the amount of residual motion. The PCA was conducted using Gromacs 2021.5 for the complexes constructed by multi-epitope vaccines with B7-1, B7-2, TLR-2, TLR-4, and projected PC1 and PC2 into two dimensions^51^.

### In silico cloning

We used the online analysis software of the SMS2 Nanjing Tate Sacrament Mirror (http://www.detaibio.com/sms2/protein_mw.html) to back-translate the amino acidsequences of multi-epitope vaccines with the codon table of invertebrate baculovirus^52^. Then, gene codon optimization was performed using the Vectorbuilder vector home server and the codon adaptation index (CAI), and GC values of the optimized codons were measured. Dual enzymatic sites were used to select the restriction endonucleases EcoRI and HindIII. Finally, the gene sequence of the multi-epitope vaccine was inserted into the pFastBac1 (insect baculovirus expression system) vector using the SnapGene tool^53^.

### Ethical approval

This study does not involve in ethical approval.

## Results

### Sequences of MOMP and CTLA-4 protein analysis

**Figure 2:**
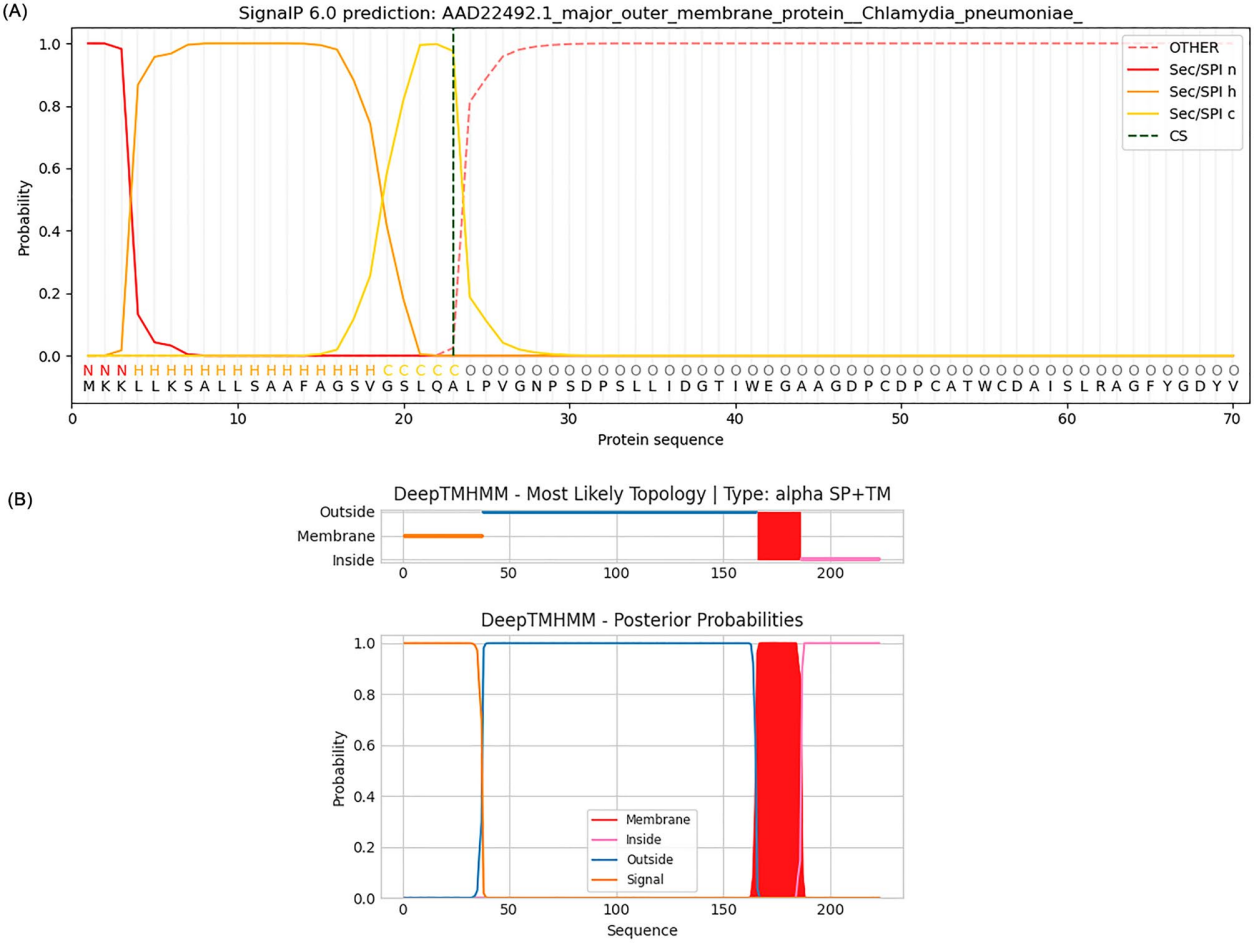
Protein signal peptide prediction (**A**). Prediction of major outer membrane protein signal peptide of C. pneumonia. The major outer membrane protein has signal peptide sequences. CTLA-4 extracellular domain analysis (**B**). The blue part indicates the extracellular domain.

The amino acid sequence of the major outer membrane protein (MOMP) (AAD22492.1) and CTLA-4 protein (NP_005205.2) was obtained from the NCBI database. The results are shown in Table 1.The signal peptide prediction of MOMP protein was performed according to SignalP 6.0 Server software, and the results showed a signal peptide at amino acids 23–24, indicating that the protein can be secreted to different cells for expression. This result is shown in Fig. 2(A).The extracellular structure prediction of CTLA-4 by DeepTMHMM-2.0 software revealed that the extracellular structural domain of human CTLA-4 is located at amino acids 38–165, as shown in Fig. 2(B).

**Table 1:** The amino acid sequence of the major outer membrane protein (MOMP) and CTLA-4 protein.

Protein	Amino acid sequence
Major outer membrane protein (MOMP) (AAD22492.1)	MKKLLKSALLSAAFAGSVGSLQALPVGNPSDPSLLIDGTIW
	EGAAGDPCDPCATWCDAISLRAGFYGDYVFDRILKVDAPK
	TFSMGAKPTGSAAANYTTAVDRPNPAYNKHLHDAEWFTNA
	GFIALNIWDRFDVFCTLGASNGYIRGNSTAFNLVGLFGVKGT
	TVNANELPNVSLSNGVVELYTDTSFSWSVGARGALWECGCA
	TLGAEFQYAQSKPKVEELNVICNVSQFSVNKPKGYKGVAFPL
	PTDAGVATATGTKSATINYHEWQVGASLSYRLNSLVPYIGVQ
	WSRATFDADNIRIAQPKLPTAVLNLTAWNPSLLGNATALSTTD
	SFSDFMQIVSCQINKFKSRKACGVTVGATLVDADKWSLTAEA
	RLINERAAHVSGQFRF
CTLA-4 protein (NP_005205.2)	MACLGFQRHKAQLNLATRTWPCTLLFFLLFIPVFCKAMHVAQ
	PAVVLASSRGIASFVCEYASPGKATEVRVTVLRQADSQVTEVC
	AATYMMGNELTFLDDSICTGTSSGNQVNLTIQGLRAMDTGLY
	ICKVELMYPPPYYLGIGNGTQIYVIDPEPCPDSDFLLWILAAVSS
	GLFFYSFLLTAVSLSKMLKKRSPLTTGVYVKMPPTEPECEKQFQ
	PYFIPIN

### T and B-Epitope prediction and screening

We predicted the B-cell linear epitopes of MOMP protein by BCPREDS and ABCpred to improve the accuracy of prediction; the common sequence measured by both software was chosen as the result of dominant epitopes of MOMP protein. Finally, four B-cell linear epitopes of this protein were screened (37–48, 63–73, 86–97, 328–339). Next, we predicted the CTL epitopes of the MOMP protein by EpiJen and NetCTLpan, and four overlapping sequences were screened as the CTL epitopes of the protein (182–189, 232–239, 238–245, 277–284) based on the prediction results of the two software. The T-cell epitopes of MOMP protein were predicted by NetNHCIIpan and SYFPEITHI, and the two software predictions were selected to filter out three common sequences as T-cell dominant epitopes of the protein (68–81, 80–93, 364–377). The above epitopes are listed in Table 2. Four B-cell linear epitopes, three T-cell epitopes, and four CTL epitopes were screened for MOMP protein. Among these epitopes, amino acids at positions 68–73 in both B-cell linear epitopes and T-cell epitopes constitute highly dominant epitopes of MOMP proteins. Furthermore, a conformational epitope with a score > 0.800 was screened as the dominant type for this MOMP protein from the software prediction results (4–21). The results are shown in Table 3.

**Table 2:** Prediction of dominant T and B-cell epitopes of the major outer membrane protein (MOMP).

Epitopes	Methods	Location	Sequence
B cell	BCPREDS, ABCpred	37–48	DGTIWEGAAGDP
		63–73	AGFYGDYVFDR
		86–97	GAKPTGSAAANY
		328–339	STTDSFSDFMQI
HTL	NetNHCIIpan, SYFPEITHI	68–81	DYVFDRILKVDAPK
		80–93	PKTFSMGAKPTGSA
		364–377	ADKWSLTAEARLIN
CTL	EpiJen, NetCTLpan	182–189	LYTDTSFS
		232–239	SVNKPKGY
		238–245	GYKGVAFP
		277–284	PLNSLVPY

**Table 3:** Prediction of conformational B-cell epitopes.

Residues	Location	Score
LLKSALLSAAFAGSVGAL	4–21	0.843

### Vaccine design, secondary and tertiary structure prediction, refinement, and validation

**Figure 3:**
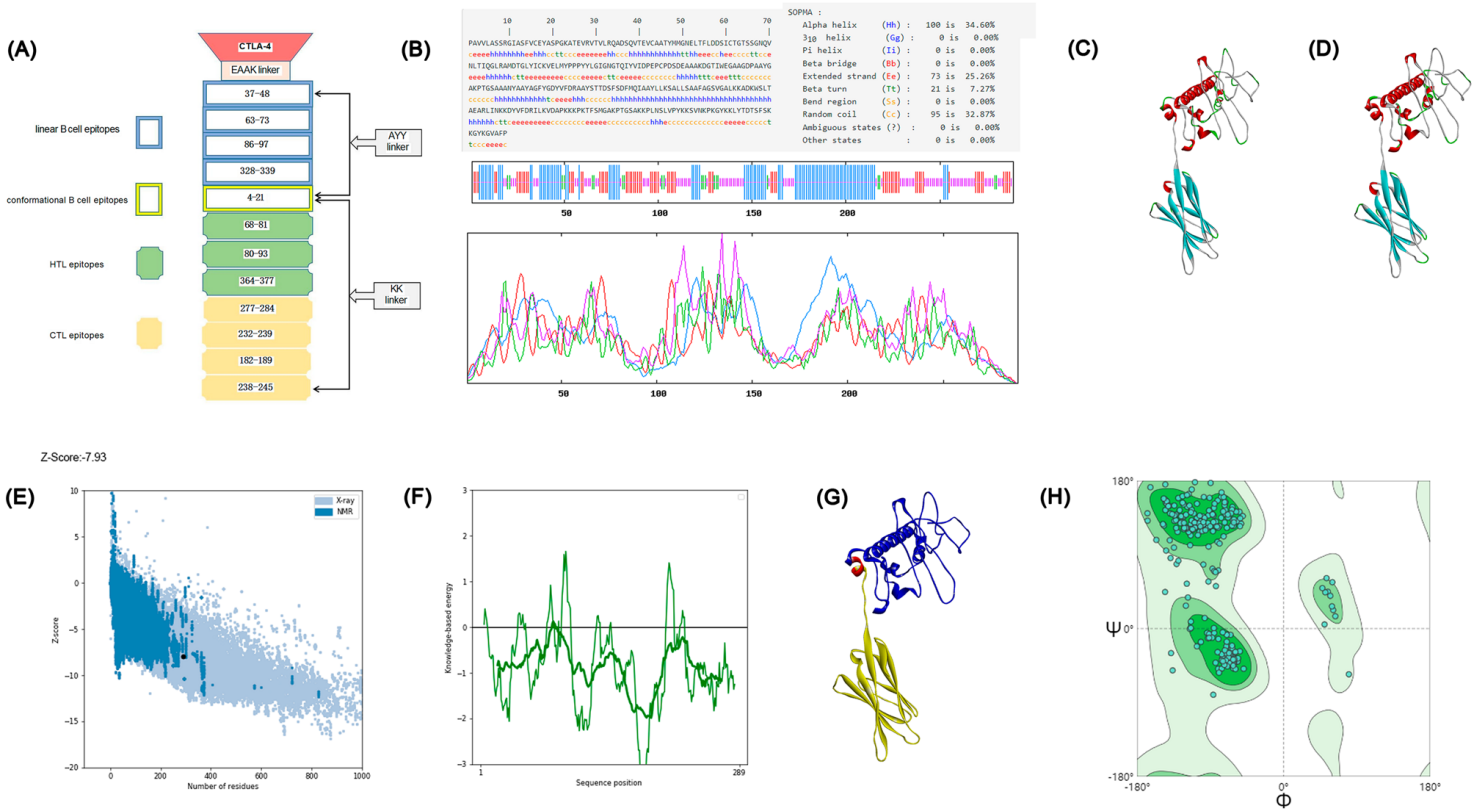
The final structure diagram of the vaccine. (**A**) CTLA-4 extracellular domain (red), linear B cell epitopes (blue), conformational B cell epitopes (bright yellow), HTL epitopes (green), and CTL epitopes (yellow). Prediction of secondary structure (**B**). Alpha helix, extended strand, beta-turn, and random coil accounted for 34.60%, 25.26%, 7.27%, and 32.87%, respectively. 3D vaccine models. (**C**) Original 3D model built by trRosetta server. (**D**) Final model refined using GalaxyRefine server. Gray represents random coil, cyan represents beta-sheet, green represents beta-turn, and red represents alpha-helix. Validation of 3D models (**E**–**H**). (**E**) The tertiary structure of the vaccine was verified by Prosa-Web, and the Z-score was − 7.93; (**F**) Plot of residue scores; (**G**) The blue part was the region of CTLA-4 extracellular domain, the red part was the region of EAAAK linker, and the yellow part was the region of the main sequence; (**H**) According to SWISS-MODEL, 96.52% of amino acid residues were in the favored regions of the Ramachandran plot.

Based on the ability of the extracellular structure of CTLA-4 to enhance the immune response induced by multi-epitope antigens, we constructed a multi-epitope vaccine by combining the extracellular structural domain of CTLA-4 and MOMP dominant epitopes were screened out by the above results. The final vaccine was composed of four parts: CTLA-4 extracellular structure, B-cell (linear and conformational epitopes), T-cell epitopes, and CTL epitopes. For the stability of the vaccine, we placed the extracellular structure of CTLA-4 at the N-terminal end of the vaccine, and the extracellular structural domain of CTLA-4 was connected to the B-cell linear epitope of the protein through the EAAK link, between the B-cell linear epitope and between the B-cell linear epitope and the conformational epitope through AYY link, the B-cell conformational epitope was connected to the T-cell epitope through KK link, and between the T-cell epitope and the CTL cell epitope through KK link; the final vaccine results are shown in Fig. 3(A).

Then, according to the analysis of the biochemical properties of the vaccine by the software ProtParam. The results showed that the vaccine consisted of 289 amino acids and had a molecular weight of 30.9 kDa (< 100 kDa), indicating that the vaccine was suitable for development. The theoretical PI value was 8.96, and the half-life in mammals was > 20 h, and the instability index was 21.41 (< 40), indicating that the vaccine is stable. The lipid index was 73.39, and the GRAVY index was − 0.171, indicating that the vaccine is hydrophilic and interacts strongly with water molecules. We also analyzed the antigenicity of the vaccine using the software VaxiJen 2.0, which showed 0.4383 > 0.4, indicating that the vaccine is antigenic. The allergenicity of this vaccine was non-allergenic according to the analysis of the software AllerTOP 2.0, indicating that the vaccine is non-allergenic.

We used the Prabi server to predict the secondary structure of the multi-epitope vaccine, and the results are shown in Fig. 3(B), which revealed that the alpha helix of this vaccine was 34.60%, extended strand was 25.26%, beta-turn was 7.27%, and random coil was 32.87%. Yanglab online software was used to construct the tertiary structure of the multi-epitope vaccine; the tertiary structure of the vaccine was selected based on Z-score, as shown in Fig. 3(C). The optimized tertiary structure of the constructed vaccine was selected by GalaxyRefine software, and finally, the tertiary structure of the vaccine was screened for model 1 based on GDT-HA, root mean square deviation (RMSD), MolProbity, Clash score, and other indicators as shown in Fig. 3(D).

To improve the accuracy of the constructed tertiary structure of multi-epitope vaccines, we used ProSA-Web and SWISS-MODEL online software to validate the constructed tertiary structure of multi-epitope vaccines. The Z-Score result using ProSA-Web prediction software was − 7.93, and in SWISS-MODEL software simulation, Ramachandran favored was 96.52%, Ramachandran outliers was 0.35%, and the results are shown in Fig. 3(E–H).

### Molecular docking and molecular dynamics simulation

**Figure 4:**
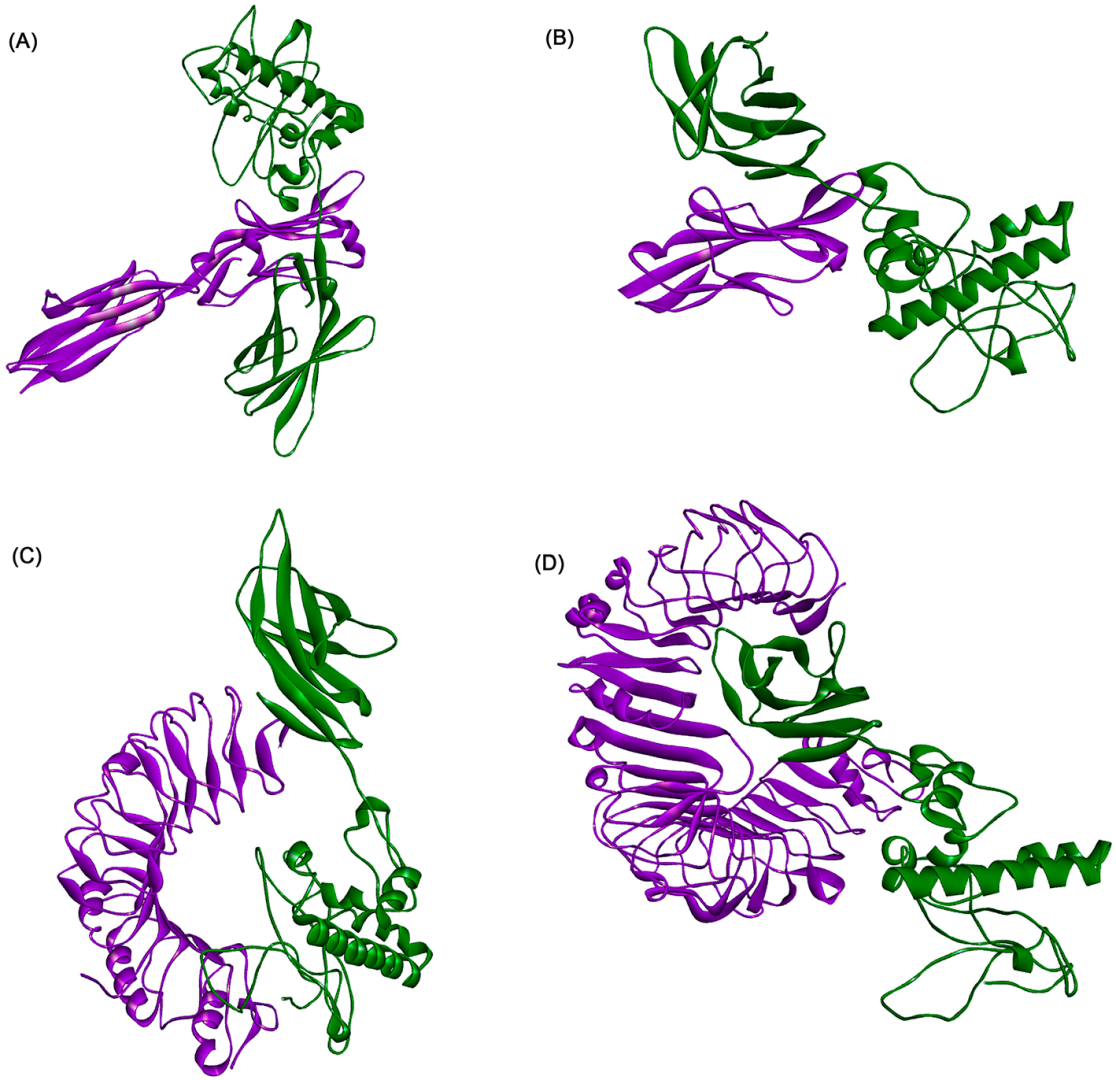
Fine docking complexes of B7-1, B7-2, and TLR-2, 4 with vaccine structure. (**A**) B7-1, (**B**) B7-2, (**C**) TLR-2, and (**D**) TLR-4. Green indicated vaccine structure and purple showed each receptor.

**Figure 5:**
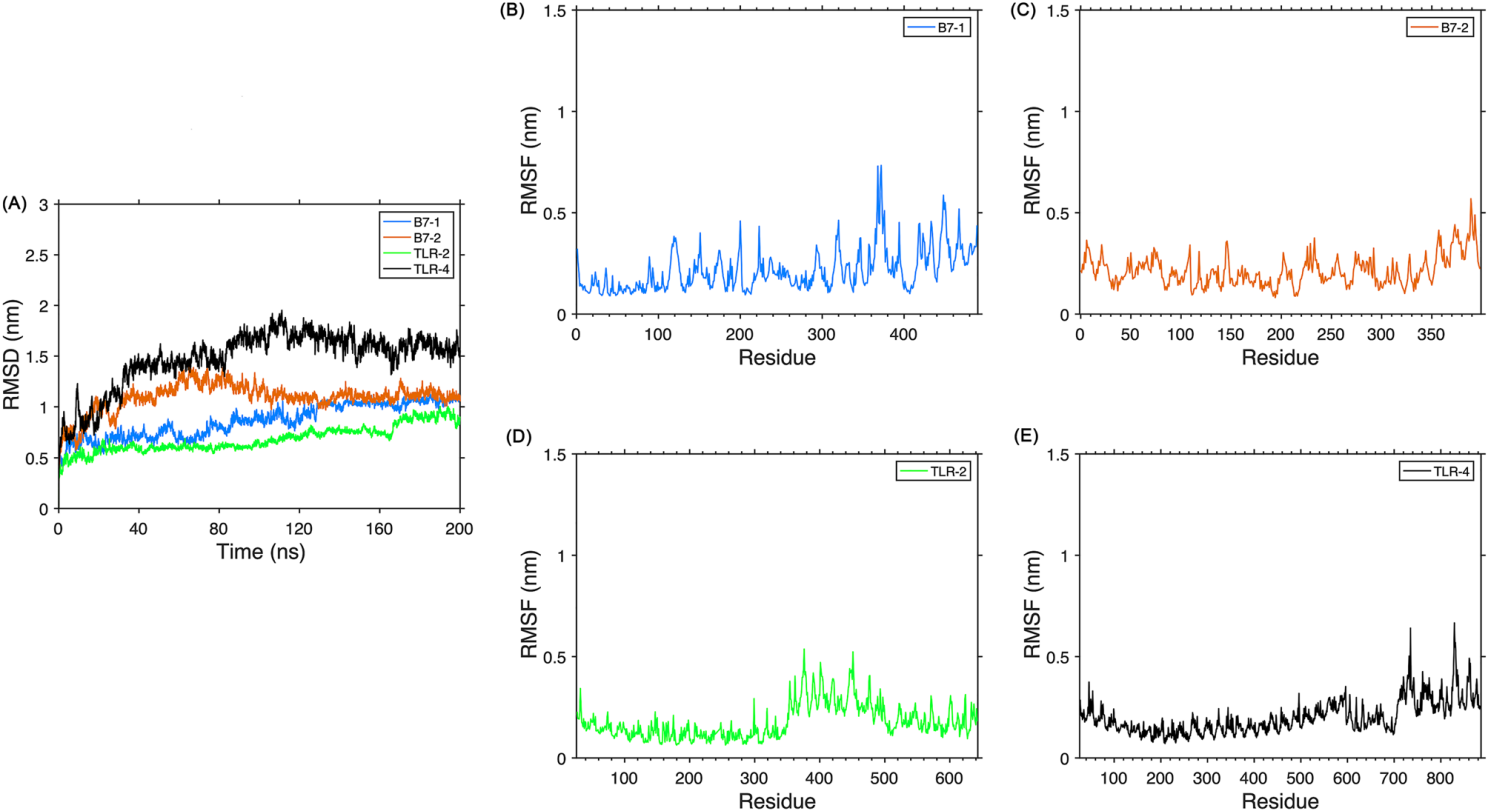
Trajectory analysis of vaccine-receptor docking complexes and conducted a simulation. (**A**) Root-mean-square deviation plots, fluctuations within 2 nm demonstrated the stability of the complexes, (**B**–**E**) root-mean-square-fluctuation plots.

**Figure 6:**
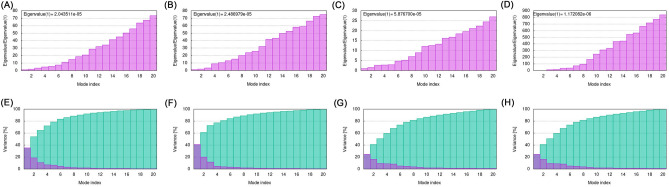
The eigenvalue plot represents the complexes formed by the combination of vaccines with (**A**) B7-1, (**B**) B7-2, (**C**) TLR-2, and (**D**) TLR-4. The smaller the value, the stronger the deformability of the complex. The variance plot represents the complexes formed by the combination of vaccine with (**E**) B7-1, (**F**) B7-2, (**G**) TLR-2, and (**H**)TLR-4. The variance plot shows the cumulative variance in green and the individual variance in purple.

**Figure 7:**
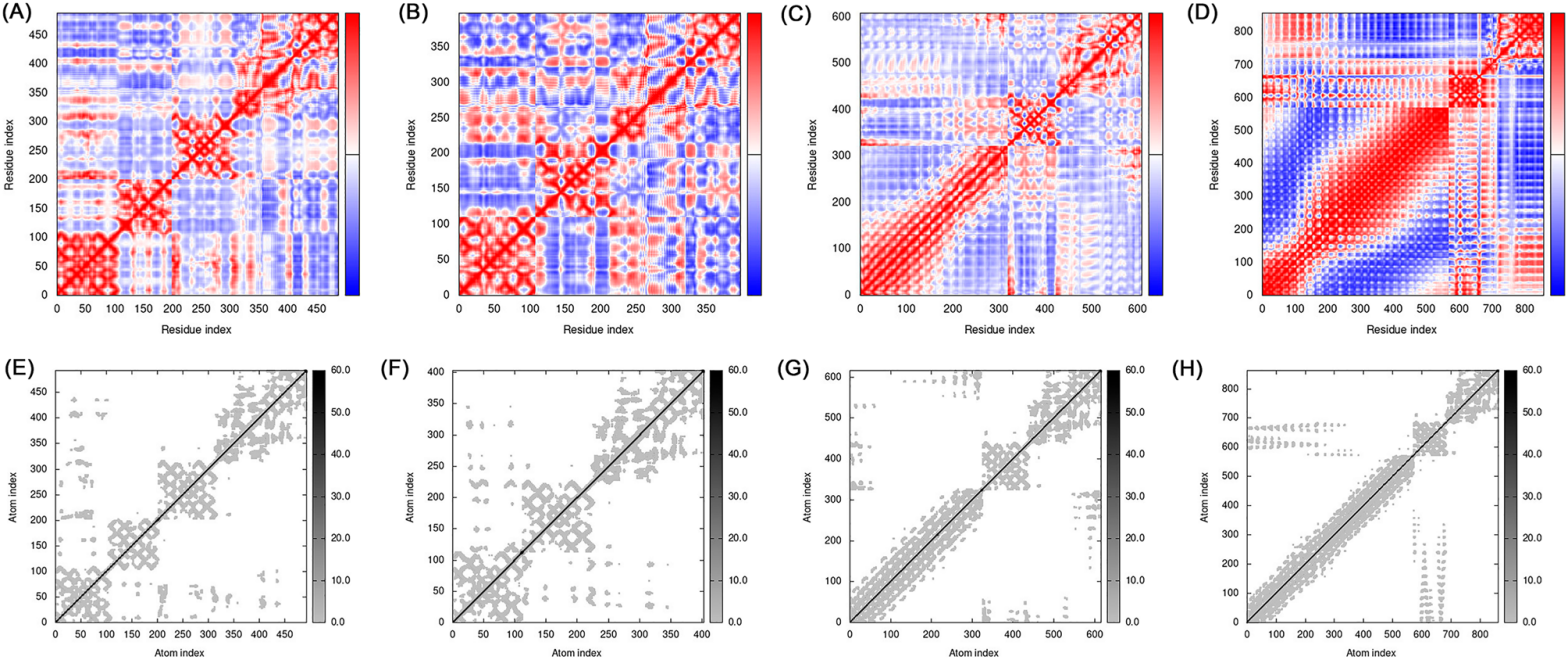
The covariance matrix represents the complexes formed by the combination of vaccines with (**A**) B7-1, (**B**) B7-2, (**C**) TLR-2, and (**D**) TLR-4. Correlated, uncorrelated, and anti-correlated motions are represented by red, white, and blue. Elastic network represents the complexes formed by the combination of the vaccine with (**E**) B7-1, (**F**) B7-2, (**G**) TLR-2, and (**H**) TLR-4. The magnitude of the interaction is proportional to the gray gradient.

We used the online software LZerD Web Server to molecularly dock the constructed multi-epitope vaccines to B7-1, B7-2, TLR-2, and TLR-4. We selected the most stable model based on the rank-sum scores (Table 4, Fig. 4). The molecular dynamics simulations of the constructed vaccines docked with B7-1, B7-2, TLR-2, and TLR-4 were performed by the software Gromacs 2021.5. The stability of the four complexes was analyzed by the root mean square deviation (RMSD). The results were that the time required for B7-1, B7-2, TLR-2, and TLR-4 complexes to reach equilibrium was 130, 60, 170, and 100 ns, with B7-2 reaching equilibrium faster than B7-1 and TLR-4 reaching equilibrium faster than TLR-2. GROMACS molecular dynamics simulations analyzed whether the system reaches equilibrium by the RMSD value, and the RMSD result within 2 nm indicates that the system is stable, and the RMSD values of the four complexes are all within 2 nm according to the analysis results, indicating that the four complex systems are stable. The root mean square fluctuation (RMSF) measures the fluctuation distance (nm) of each amino acid residue relative to the equilibrium site during the simulation, and the results indicate a high degree of flexibility in specific regions, as shown in Fig. 5. In addition, we also analyzed the stability and flexibility of vaccine complexes with B7-1, B7-2, TLR-2, and TLR-4 proteins by iMODS, and the results of the analysis are shown in Figs. 6 and 7, with eigenvalues of 2.043511e^-5^, 2.486979e^-5^, 5.876700e^-5^, and 1.172082e^-6^, indicating that these four complexes have strong deformability. The above results indicated that the four composite systems have stability and flexibility.

**Table 4:** Molecular docking final model score.

Name	GOAP Score	GOAP Rank	DFIRE Score	DFIRE Rank	ITScore score	ITScore Rank	Score Ranksum
B7-1	− 55,064.83	97	− 39,065.23	8	− 20,099.91	3	108
B7-2	− 42,068.43	95	− 31,808.18	28	− 16,387.11	22	145
TLR-2	− 77,519.01	371	− 54,220.83	211	− 26,515.34	107	689
TLR-4	− 114,074.97	1648	− 81,561.68	343	− 39,876.3	131	2122

### Principal component analysis

**Figure 8:**
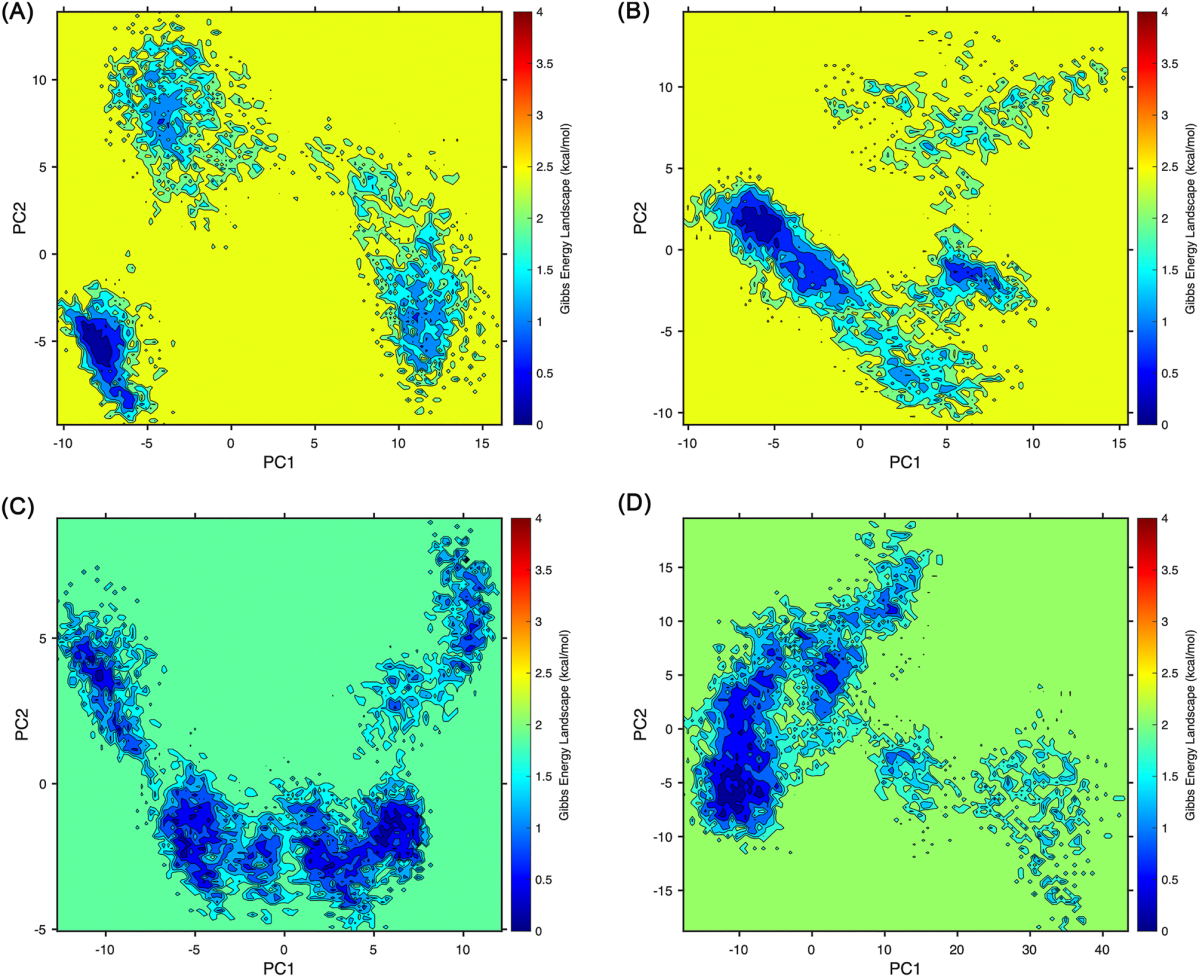
Projection of trajectories into PC1 and PC2 for the docking complexes. (**A**) B7-1-vaccine complex, (**B**) B7-2-vaccine complex, (**C**) TLR-2-vaccine complex, (**D**) TLR-4-vaccine complex.

We analyzed the major component analysis of this vaccine in complex with B7-1, B7-2, TLR-2, and TLR-4 by GROMACS 2021.5. PCA showed conformational differences between different systems, and the results indicated (Fig. 8) that the PC1 of B7-1 is in the range of -10-14 nm, PC2 is in the range of -10-15 nm, the PC1 of B7-2 is in the range of -10-14 nm, PC2 is in the range of -10-10 nm, the PC1 of TLR-2 is in the range of -13-11 nm, PC2 is in the range of -5-9 nm, and the PC1 of TLR-4 is in the range of -15-40 nm, PC2 is in the range of -10-19 nm, indicating that the four systems have some stability. The range of PC1 of TLR-4 is greater than that of the other systems, indicating that the stability of this systems is weaker than the others system, which is more consistent with the RMSD value analysis.

### In silico cloning

**Figure 9:**
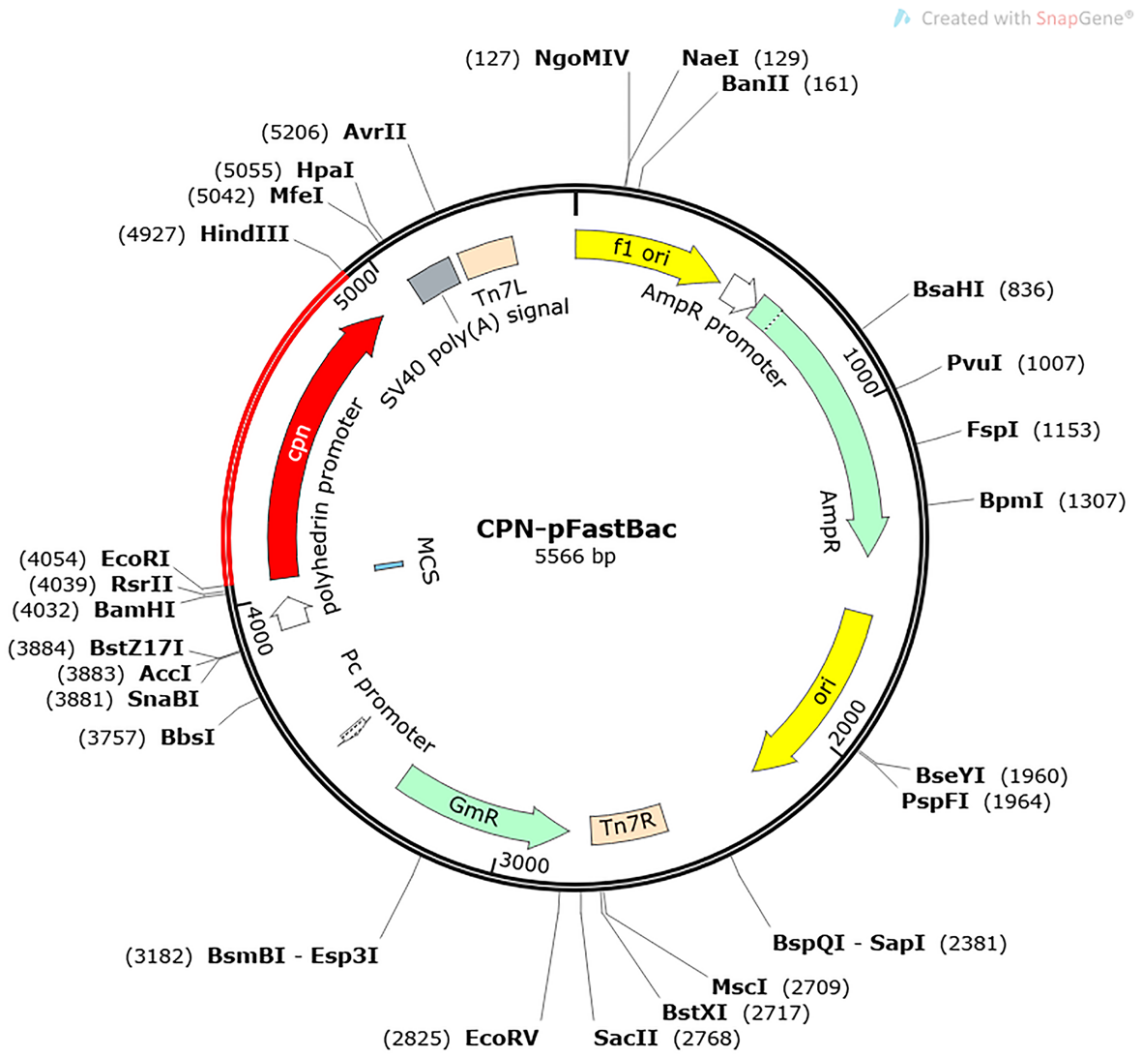
The structure of the in silico vector pFastBac1, including the vaccine, vector, and restriction sites. The red region represents the vaccine-encoding gene.

We used SMS2 Nanjing Tide BioMirror online prediction software for reverse translation of multi-epitope vaccine structures and set the codon usage table to baculovirus. The results of back-translation by VectorBuilder vector were optimized, 867 nucleotides were input, Sodoptera frugiperda was selected, and the GA and GC values of the gene sequences were evaluated. The optimized results showed a CAI value of 0.93 (the ideal value is 0.8–1.0; the higher the number, the less likely the gene is poorly expressed) and a GC content of 58.02% (the optimal value is 30–70%). Finally, the optimized gene sequence was inserted into the pFastBac1 vector for expression using SnapGene software (Fig. 9). These results indicate that the proposed vaccine construct can be efficiently expressed in the pFastBac1 vector.

## Discussion

The *C. pneumoniae* infection is prevalent in humans, and because it invades the body and causes monocyte-macrophage reactions, alveolar macrophages act as carriers of pathogen storage and transmission, resulting in persistent infection in the host and because the current treatment of the infection mainly uses antibiotics, which can easily lead to antibiotic abuse, a vaccine that can effectively prevent the disease is essential. Incontrast to conventional vaccines, we used immunoinformatics technology to construct multi-epitope vaccines that can be expressed on insect baculovirus vectors. These vaccines can effectively and safely prevent *C. pneumoniae* infection, thereby proposing novel ideas for the development of related vaccines.

First, we selected the candidate antigen through a literature search, MOMP protein, which is the main component of the extra-membrane protein of *C. pneumoniae* and is highly conserved and immunogenic. Next, we predicted the signal peptide of this protein using SignalP 6.0 server software, which revealed a signal peptide of amino acids 23–24, indicating that the protein can be targeted and located outside the cell. Second, while constructing the multi-epitope vaccine, we predicted and screened four B-cell linear epitopes, one conformational B-cell epitope, three T-cell epitopes, and four CTL epitopes of MOMP protein by online software. In order to improve the targeting of APC and enhance the immune response, we added the extracellular structural domain of CTLA-4 at the N-terminal end of the vaccine protein sequence and connected the predicted four epitopes by EAAK link and between the four epitopes using the AYY link and KK link, and then constructed the multi-epitope vaccine.

In order to determine whether the multi-epitope vaccine can be expressed safely and effectively, we assessed a series of predictions and expressions of the vaccine in vectors. The biochemical properties, antigenicity, and allergenicity of the vaccine were evaluated by online software, the results showed that the vaccine consists of 289 amino acids and a molecular weight of 30.9 kD and an antigenic score of 0.4383 (> 0.4 indicates antigenicity). The allergenicity of the vaccine was predicted to be non-allergenic, indicating that the vaccine is non-allergenic. Then, we predicted the secondary structure of the multi-epitope vaccine using Prabi server, predicted and optimized the tertiary structure of the vaccine using Yanglab and GalaxyRefine, and verified the tertiary structure using Prosa-Web and SWISS-MODEL with a Z-score of − 7.93; the negative value indicates a high probability of correctly predicting the tertiary structure.

In order to evaluate the interaction between this vaccine and B7-1, B7-2, TLR-2, and TLR-4, we used LZerD Web Server for docking and subjected the docked complexes to MD simulations and PCA to analyze the stability of their complexes; the results indicated that all four systems are stable. Since the production of multi-epitope vaccines requires a suitable heterologous system for expression, we chose the insect baculovirus expression system as the source of heterologous expression. Anti-translation and codon optimization of the multi-epitope vaccine based on the codon table of baculovirus predicted a CAI of 0.93 and a GC value of 58.02%, indicating that the multi-epitope vaccine is promising for expression on insect baculovirus vectors. Finally, the optimized gene was cloned in silico on the pFastBac1 vector system.

Currently, all clinical trials of *C. pneumoniae* have failed^54^. Researchers are starting to develop a number of *Chlamydia pneumoniae* therapeutics, such as employing immunoinformatics to screen for new medicines that predominantly target *Chlamydia pneumoniae* infections. When we compare the results with previous studies, it is noteworthy there is a epitope-based *malaria* vaccine to enter phase III clinical trials and has the potential to be the first licensed vaccine against a human parasitic disease^55^. A number of epitope-based vaccinations are also set to undergo phase II and phase III clinical trials in the future years^56^. As a result, multi-epitope vaccinations might become an essential weapon in the battle against illnesses in the future. It should be emphasized that the experiment on the immunogenicity and protective effect of *C. pneumoniae* vaccine is currently being implemented, and the relevant results will be reported in subsequent research report.

## Conclusion

In this study, a safe and effective multi-epitope vaccine was designed using an immunoinformatics approach for the prevention of *C. pneumoniae* infection. First, we identified the amino acid sequences of MOMP and CTLA-4 to further predict the extracellular structural domain of CTLA-4. Also, the B-cell linear epitope, conformational B-cell epitope, T-cell epitope, and CTL epitope of the MOMP protein were predicted and screened. Then, a multi-epitope vaccine containing the extracellular structural domain and four epitopes of CTLA-4 was constructed, and the safety and stability of the vaccine were determined by predicting the biochemical properties and spatial conformation of the vaccine. The interaction of the vaccine with B7-1, B7-2, TLR-2, and TLR-4 was analyzed by MD simulation and PCA analysis. Finally, in silico cloning on the pFastBac1 vector system showed that the constructed multi-epitope vaccine had good expression prospects on the pFastBac1 vector system. In conclusion, the current results provided novel strategies for developing *C. pneumoniae* vaccines; however, future experimental validation of this vaccine candidate is needed to determine its efficacy, effectiveness, and safety.

## Data Availability

Data is with the authors and will be provided on request through corresponding author. The proteome sequences of C. pneumoniae (AAD22492.1) were downloaded from UniProt (https://www.uniprot.org/). The human CTLA-4 amino acid sequence (NP_005205.2) was obtained from the National Center for Biotechnology Information (NCBI).
